# An etiopathogenesis of juvenile idiopathic arthritis: the protein-homeostasis-system hypothesis

**DOI:** 10.3389/fped.2026.1698713

**Published:** 2026-01-29

**Authors:** Kyung-Yil Lee, Jung-Woo Rhim

**Affiliations:** 1 Department of Pediatrics, College of Medicine, The Catholic University of Korea, Seoul, Republic of Korea; 2 Junglock Biomedical Institute, Deajeon, Republic of Korea

**Keywords:** epidemiology, etiology, juvenile idiopathic arthritis, pathophysiology, protein-homeostasis-system hypothesis

## Abstract

The initiation of juvenile idiopathic arthritis (JIA) may be associated with an infection caused by unidentified pathogens. The prevalence or incidence rates of JIA differ markedly among populations. The constituent of microbiota of human species is influenced by age during childhood and differs among ethnic groups. On occasion, some strains in microbiota can invade the host and elicit inflammatory immune reactions, and dysbiosis has been observed in JIA. The microbial-infected cells contain inflammation-inducing substances including pathogen-origin substances such as toxins and pathogen-associated molecular patterns and host cell-origin substances such as damage-associated molecular patterns, biochemicals, and pathogenic proteins/peptides. The immune systems of mammals, especially adaptive immune system, mature along with ages in childhood and decline in old age. JIA has epidemiological and clinical characteristics including different incidence by ethnic groups with similar age and sex predilection in certain subtypes, an association with various infectious and immune-mediated diseases and physical trauma, and a different clinical nature as compared with arthritis in adults. Here, it is proposed that causal agents of JIA are certain strains in microbiota, and etiological or inflammation-inducing substances in JIA are derived from the infected or injured cells through the characteristics of JIA and the PHS hypothesis. Patients with JIA may have an immature or improper adaptive immune state for controlling of the substances.

## Introduction

Juvenile idiopathic arthritis (JIA) is a generic term for arthritis that has an onset below the age of 16 years, persists for more than six weeks and consists of heterogeneous clinical phenotypes with age and sex predominance in certain subtypes (1). Adults are also affected with arthropathies having JIA criteria with rare frequencies, suggesting that a similar pathogenesis is involved in similar types of arthritis in both children and adults. However, clinical, pathological, and laboratory findings are somewhat different in nature between children and adults (2, 3).

Incidence or prevalence rates of JIA, including subtypes, are variable across ethnic groups or populations in contrast to those observed in the case of common infectious diseases (4).

The ecologic system of microbes of the host, that is microbiota, is associated with a disordered state of the host, and dysbiosis has been reported in various immune-mediated diseases including JIA (5). The profile of microbiota is influenced by age during childhood and differ in populations due to variations in environmental factors including diet and socioeconomic status (6).

JIA has been suggested to be associated with an infection of various pathogens, though the pathophysiology of JIA remains to be fully elucidated (7, 8). Today, it is known that the immune system of the host responds not only to substances that originate from pathogens such as toxins and pathogen-associated molecular patterns (PAMPs), but also to substances that originate from injured host cells including damage (danger)-associated molecular patterns (DAMPs) (9). Several DAMPs or alarmins, including high mobility group box 1 (HMGB 1), S100 proteins, and heat shock proteins (HSPs) have been reported to be associated with the pathogenesis of JIA (10). The diseases, including infectious diseases, Kawasaki diseases (KD), multisystem inflammatory syndrome in children (MIS-C), and JIA have etiological or inflammation-inducing substances, and a disease state begins when etiological substances bind to affinitive receptors on target cells in organs. The protein-homeostasis-system (PHS) hypothesis proposed that the immune system of the host control the toxic substances for protection of the cells, and each component in immune systems controls them according to the size and biochemical property (11–13). In this article, we discuss unsolved issues surrounding the epidemiological, clinical, and immunological aspects of JIA and propose a new concept for the ethology and pathophysiology of JIA based on its clinical and epidemiological characteristics and the PHS hypothesis.

## Epidemiology in JIA

Since JIA was first reported in the literature during the late 19th century (14), JIA has been found to occur around the world. The International League of Associations for Rheumatology (ILAR) designated seven subtypes as follows: 1) systemic JIA; 2) oligoarthritis (persistent/extended); 3) rheumatoid factor (RF)-negative polyarthritis; 4) RF-positive polyarthritis; 5) psoriatic arthritis; 6) enthesitis-related arthritis (ERA); and 7) undifferentiated arthritis (15). Although JIA is a relatively rare disease and there is a lack of resources in many countries, epidemiological studies have reported that prevalence or incidence rates vary among populations. In developed countries, estimated prevalence rates range from 3.8 to 400 cases per 100,000 children, and incidence rates range from 1.6 to 23 per 100,000 children (4), being lowest in a Japanese study (less than 1 per 100,000) and highest in a Norwegian study (71 per/100,000) (16, 17). The pooled incidence estimates for individual JIA subtypes were reported as 0.5 for systemic JIA, 4.5 for oligoarthritis, 0.7 for RF-negative polyarthritis, 0.5 for RF-positive polyarthritis, 0.4 for psoriatic arthritis, and 2.0 for spondyloarthropathies including ERA per 100,000 children, respectively (18). JIA appears more commonly in Western countries vs. in East Asian countries, including Japan and South Korea, opposite to incidence of KD. Contrast to JIA, the prevalence and incidence rates of rheumatoid arthritis (RA) in adults show less discrepancy across the populations with geographical and time-based variations (19). In medical history, symmetrical erosive peripheral polyarthritis, as a phenotype of RA as well as JIA might be absent or very rare in Western countries prior to 18th century, which had been relatively common in native Indians in New World at the Colombian era. It suggests that this type of RA is also associated with unidentified pathogens and gradually spreads over the world (20). Variations in incidence rates among ethnic groups have been observed in other acute or chronic infection-related immune-mediated diseases including KD and MIS-C, and inflammatory bowel disease (IBS) and Behçet's disease (21). These findings have been suggested that ethnicity, genetic susceptibility, or environmental factors are involved in the pathogenesis of JIA as well as KD. While the prevalence of common infectious diseases in childhood such as exanthem subitum, influenza, and respiratory syncytial virus infection may be similar among populations, but epidemiological characteristics of JIA and KD, such as age and sex predominance, and no appearance of seasonal and periodic epidemics suggests that infectious agents and pathophysiology of JIA are distinct to those in common infectious diseases (22).

## Pathogenic conditions associated with arthritis and possibly JIA

Because JIA is a childhood disease as with common infectious diseases, an infection caused by unidentified pathogens may be associated with disease onset. In children, postinfectious arthritis is not rare, which is mostly transient and self-limited. However, a small part of patients with postinfectious arthritis can develop chronic arthritis or JIA, suggesting that infection is the most important etiology of JIA (7, 8). Viruses such as rubella virus, measles virus, influenza A and B viruses, and parvovirus B19 have been suggested to be linked with the onset of JIA (23–26). During the coronavirus disease 2019 (COVID-19) pandemic, there have been case reports on chronic arthritis or JIA after severe acute respiratory syndrome coronavirus-2 infection (27). Also, bacteria including *Mycoplasmas*, Group A streptococcus, *Salmonella* species, *Borella burgdorferi* for Lime disease (28–30), and other pathogens such as chlamydia and rickettsia (31, 32) have been suggested to be associated with the onset or exacerbation of oligo/polyarticular JIA. Post-vaccination arthritis after inoculation of live or killed vaccines, including rubella, influenza, and hepatitis B vaccines, has been described as an entity of chronic arthropathy (33–35). Also, cases of chronic arthritis after vaccination against COVID-19 have been reported (36). An entity of postinfectious arthritis, that is, reactive arthritis (ReA) is identified. ReA affects mainly young adults who have an aberrant or hyperactive immune function and is associated with the human leukocyte antigen (HLA)-B27 genetic marker. The most investigated triggering pathogens include *Salmonella*, *Shigella*, and *Campylobacter* in intestinal infections and *Chlamydia trachomatis* in sexually transmitted infections, but other rare pathogens can be associated with ReA (31). ReA appears approximately 1–3 weeks after a known infection, and some patients are affected with extra-articular symptoms such as conjunctivitis, urethritis, cervicitis, skin rashes, and chronic organ diseases including immunoglobulin A (IgA) nephropathy and cardiopathies (37). Some patients with chronic immune-mediated diseases, including psoriasis, systemic lupus erythematosus (SLE), IBS, Behçet's disease, and Whipple's disease (38–40), and other autoimmune disease can be affected by chronic arthritis (41–45). Arthritis is not always synchronous with those diseases’ activity and generally appears after the stage of active tissue cell injuries in each disease, suggesting that materials from injured tissue cells are associated with arthritis. Some patients with acute infection-related immune-mediated diseases such as acute rheumatic fever, KD, and Henoch-Schönlein purpura (or IgA vasculitis), experience transient arthritis, rarely chronic arthritis, during the clinical courses (46–48). Furthermore, arthritis in some patients with systemic JIA appears following the subsiding of prolonged fever and skin rashes within six months. The conditions associated with arthritis and possibly JIA are expressed in Table 1.

**Table 1:** Conditions associated with arthritis and possibly JIA (references).

Post-infectious	Non-infectious
Viruses	Chronic immune-mediated diseases
Rubella virus (23)	Psoriasis
Measles virus (24)	Systemic lupus erythematosus
Influenza viruses (25)	Inflammatory bowel disease (38)
Parvovirus B19 (26)	Behcet disease (39)
Coronaviruses (SARS-CoV-2) (27)	Whipple's disease (40)
Other viruses	Juvenile dermatomyositis (41)
	Polyarteritis nodosa (42)
Bacteria	Sarcoidosis (43)
*Mycoplasmas* (28)	Sjögren's syndrome (44)
Group A streptococci (29)	Castleman disease (45)
*Borrelia burgdoferi* (30)	
Salmonella species (31)	Acute immune-mediated diseases
Shigella species (31)	Acute rheumatic fever (46)
Campylobacter species (31)	Kawasaki disease (47)
	Henoch-Schönlein purpura (48)
Other pathogens	
Chlamydia trachomatis (31)	Post-vaccine
Rickettsia (32)	Rubella vaccine (33)
	Influenza vaccine (34)
	Hepatitis B vaccine (35)
	Covid-19 vaccine (36)

**Figure 1:**
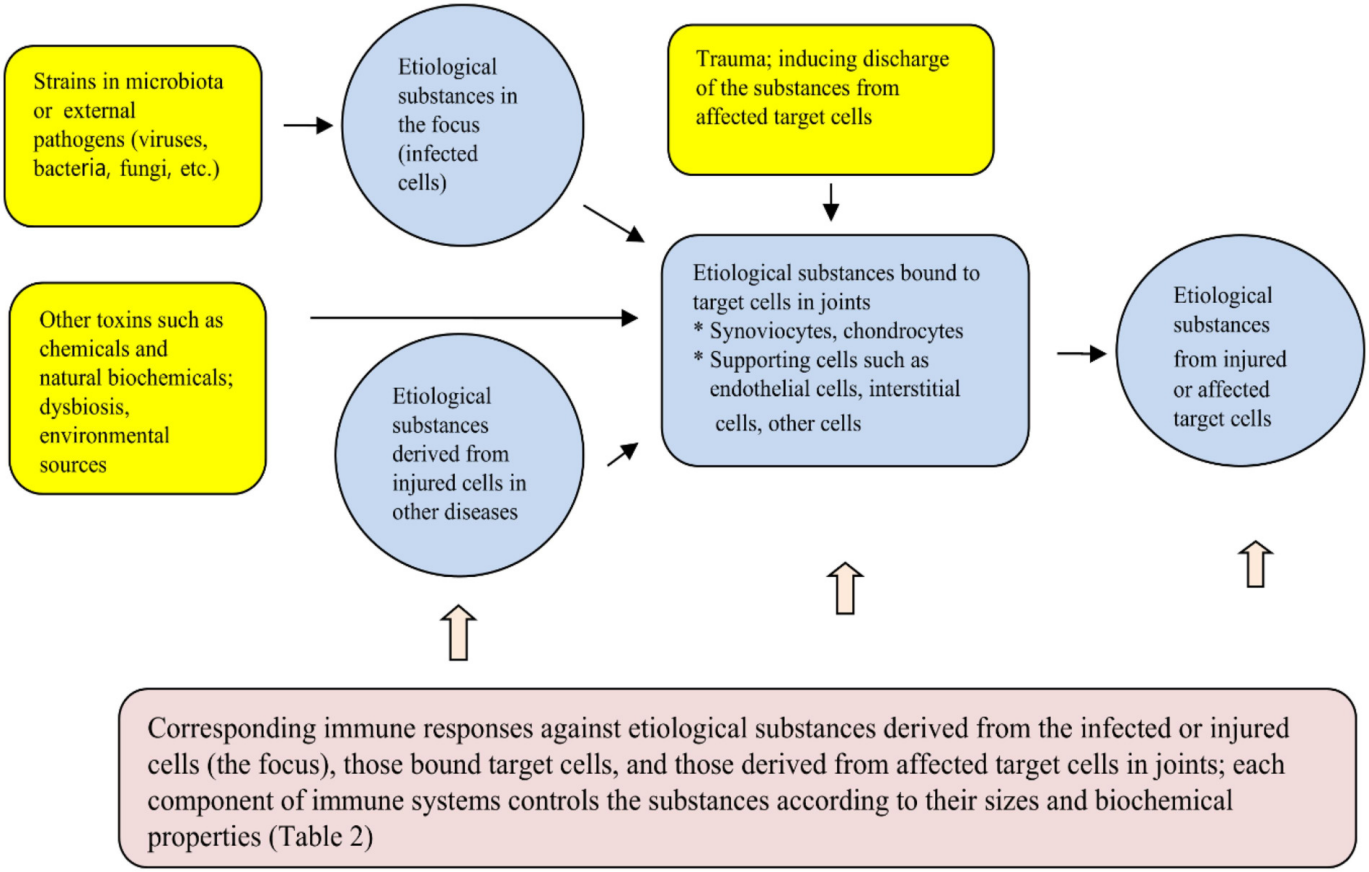
Schematic diagram of immunopathogenesis of JIA.

It is an important note that there is a difficulty to prove the pathogens through synovial fluid culture or to identify the whole pathogen or structural components of the pathogens in pathologic lesions in postinfectious arthritis, ReA, and JIA. Given that multiple pathogen infections and diverse acute or chronic immune-mediated diseases are associated with chronic arthritis or JIA, etiological or inflammation-inducing substances in these conditions may be originated from the infected or injured cells as the focuses; they include not only toxins and PAMPs from the infectious agents but also DAMPs and other bioactive substances from affected host cells. Because the kinds of etiological substances are determined by the invaded intracellular pathogens and kinds of infected cells, it is plausible that appearing of subtypes of JIA and different clinical manifestations in in each subtype are related to different pathogen species that invade same kinds of cells or same pathogens invade different tissue cells. It is believed that initial clinical manifestations, pathologic findings of arthritis, and activated immunological biomarkers are similar between postinfectious/reactive arthritis and JIA, but natural course of the disease is longer and long-term sequala is higher in JIA. Thus, clinical differences of both arthritis depend on the immune components involved in joint inflammation of the host, since immune components control the initial etiological substances targeting joint cells derived from the focuses, and the substances secondarily derived from injured target joint cells, including synovial cells, chondrocyte cells, vascular endothelial cells, connective tissue cells and periosteal cells (Figure 1).

## Microbiota and JIA

Microbiota consists of bacteria, viruses, and fungi in the upper and lower respiratory tracts, gastrointestinal tract, skin, and lower urogenital tract of the host. The components of microbiota differ in individuals and ethnic groups and can be changed according to age and environmental factors such as diet and antibiotic use (49, 50). The normal flora or commensals in the microbiota of the human species have evolved with the species and are now regarded as a critical part of the immune system (51, 52). The dysbiosis becomes an important study subject in nearly all medical fields, and intestinal dysbiosis has been reported in patients with JIA. JIA patients have reportedly more abundant bacteria belonging to genera Bacteroidetes and Actinobacteria, and Fusobacteria are present only in JIA patients compared to healthy controls (53). Different dysbiosis strains are observed in three subtypes of JIA, polyarticular JIA, ERA, and non-ERA, and different microbial profiles and intra-group variability were reported in both the active disease stage and in the remission stage (54). The mechanisms by which dysbiosis provokes the disease remain undetermined. Excessive production of toxic materials from dysbiotic microbials, vulnerability of invasion of pathogens or toxins via the impaired mucosal barriers, or disruption of the equilibrium of the microbiota affecting the host's immune system have been suggested (55, 56). On the other hand, it is well-known that on occasion, some strains in the microbiota can invade and elicit diseases. For example, pathogens of common infectious diseases such as acute pyelonephritis, and otitis media, sinusitis and pneumonia are *E. coli* or *S. pneumoniae,* respectively, that are now regarded as normal flora of human species (22). Evidence of exposure to gut bacterial products, including lipopolysaccharide (LPS) IgG antibody, LPS-binding protein, or *α*-1-acid glycoprotein in the sera of patients with JIA, has been reported (57), suggesting that certain strains in microbiota can invade the host. Given that the composition of microbiota is influenced by age, sex, and ethnic groups in conjunction with cultural environments, marked differences in racial incidence, the age predominance or the female predominance in certain subtypes of JIA may be associated with the colonization state of the etiologic agents in the microbiota. Since pathogens in microbiota are colonized first and then, invade the hosts/host cells, it is possible that the pathogens of female-dominant JIA in girls, RA, and SLE are related to the strains located in the female urogenital tract.

## Genetic, epigenetic, and environmental factors in JIA

Genetic studies for JIA have suggested that multiple genes, including HLA genes, are associated with susceptibility or phenotypes of JIA. Oligoarticular JIA has reportedly been associated with HLA-A2, HLA-DRB1*11, and HLA-DRB1*08, and polyarticular JIA reported to be associated with HLA-DR4 (58, 59). Additionally, many genes, especially immune-associated genes, including *PTPN22* and *PTPN2*, have been reported for associations with JIA (60). On the other hand, the results of genetic study are influenced by the number of subjects and may be different in populations, and there are few candidate genes as diagnostic-assistant indices, except HLA-B27 (61).

Epigenetic mechanisms that modulate gene expression have introduced to explain the link between genetic factors and acquired predisposing factors in JIA. Well-studied epigenetic factors include micro-RNAs (miRNAs), gene methylation, and long non-coding RNAs (LncRNA). miRNAs are small, non-coding, RNA molecules that bind to messenger RNAs and disrupt the transcription of target genes including immune-associated genes. Researchers have suggested that miRNAs could be used as diagnostic biomarkers and provide information on disease activity and progression. Changed or abnormal expressions of miRNAs have been reported in many inflammatory and immune-mediated diseases including JIA and RA (62, 63). Gene methylation regulates gene expression by recruiting proteins involved in gene repression or by inhibiting the binding of transcription factors to DNA. Studies have shown that hyper- or hypo-methylation of various genes is associated with RA disease activity (64). LncRNA is a newly identified non-coding RNA widely expressed in various tissues of the human body, which consists of more than 200 nucleotides in length. LncRNA may be involved in a wide range of biological activities from epigenetic regulation and chromatin remodeling to transcriptional and posttranscriptional modification. Several studies have reported dysregulation of LncRNA in peripheral blood cells or fibroblast-like synoviocytes in patients with RA (65). On the other hand, it remains unknown whether epigenetic alterations observed in the diseases are a contributing factor for development or progression of the diseases or secondary or adaptive phenomena appearing during the disease processes for immune reactions in need.

Environmental factors affecting the populations include food culture (or diet), sanitation, and socioeconomic status in conjunction with attainable medical services. For example, antibiotics (quinolone) can induce arthritis and/or enthesitis in children. Environmental conditions change over time due to economic development in communities or countries. As previously mentioned, main diet among ethnic groups constructs different microbiota, and the constituents in microbiota are changing according to ages during childhood together with maturation of immune system, especially adaptive immune system. Although the incidence of acute and chronic immune-mediated diseases is quite different among the populations, clinical manifestations of these diseases are similar, and immune function of children against insults from JIA or KD may be nearly identical across the populations. Thus, it is possible that the difference incidences in the ethnic groups and predilection of age and sex are associated with colonization states of causal agents in microbiota, and children living in higher prevalent countries may have more chances of being exposed to JIA or KD pathogens (13, 22).

## Etiological or inflammation-inducing substances of JIA

Almost all human diseases involve etiological substances for disease-onset although we cannot define all of them at present time. The toxic substances to the target cells of the host include small materials such as toxic elements and monoamines, chemicals including drugs, and biochemicals such as natural toxins from insects or snakes, pathogenic protein/peptides, and toxic or bioactive PAMPs and DAMPs. DAMPs are defined as substances produced by cells injured due to various events including infection and trauma. Well-studied intracellular DAMPs include HMGB1, S100 proteins, and HSPs. DAMPs bind to Toll-like receptors (TLRs) or intracellular sensors of innate immune cells or affected cells in a similar way as do PAMPs. Also, DAMPs are associated with the pathogenesis of immune-mediated diseases including JIA and RA (66). TLRs are expressed on synovial fibroblasts, monocytes, and macrophages from synovial fluid obtained from patients with RA, and innate immune reactions through TLR2 and TLR4, as well as endosomal TLR3, TLR7, and TLR 9, contribute to the pathogenesis of RA and subtype of JIA (67, 68). HMGB1 is a highly conserved non-histone nuclear protein that regulates chromatin structure and the transcription process through interaction with DNA (69). HMGB1 levels are increased in patients with JIA or RA and are associated with the pathogenesis of those diseases (70). The methotrexate treatment reduces HMGB1 expression in synovial tissues (71), and HMGB1 genetic polymorphism are reportedly correlated with outcomes in RA (72). S100 proteins, including calprotectin, consist of a family of calcium-binding proteins. S100 proteins are associated with clinical disease activity, inflammatory parameter values, and evaluated pathologic lesions and used clinically as a tool for the evaluation of immunological remission in JIA or RA patients (73, 74). HSPs play a cytoprotective role by modulating cell death signalling pathways and act as intracellular chaperones, and the expression of these proteins is increased and released to the extracellular space, where they perform diverse immunological functions (75). Experimental and clinical studies have reported that some kinds of bacterial or self-heat shock proteins, including HSP 60 and HSP 65, are associated with inflammation control in JIA or RA (76, 77).

It is a reasonable assumption that there are many unidentified inflammation-inducing substances derived from injured host cells. The pathogen-infected cells contain known replicating pathogens, byproducts of pathogens such as PAMPs, toxins, capsid proteins, and pathogen DNAs and RNAs. Moreover, in bacteria and fungi infections, the pathogens possess their co-living phage (viruses) in the affected focuses (78). Besides DAMPs, there are numerous products from intracellular organelle such as mitochondria, immune proteins such as interferons, antimicrobial peptides, and proteasomes with related peptides (79). On occasion, these diverse substances can release and spread systemically or locally as a form of viremia or bacteremia and bind to the target cells and induce inflammation with the corresponding immune components. For example, severe pneumonia and acquired respiratory distress syndrome (ARDS) are caused by not only pathogen-associated insults but also nonpathogen-associated insults such as blunt chest trauma, gastric content aspiration, multiple injuries, multiple transfusions, burns, pancreatitis, and amniotic fluid embolism (80). As previously discussed, patients with acute or chronic immune-mediated diseases, including acute rheumatic fever, KD, psoriasis, and SLE, can be affected by arthritis, suggesting that arthritis-eliciting substances are associated with substances derived from injured cells in each disease. Henoch-Schönlein purpura (or IgA vasculitis) is a subacute, self-limited, infection-related immune-mediated vasculitis in childhood though adults can also be affected rarely. Purpuric vasculitis in skin appears mainly in pressured areas such as the buttocks and lower extremities and can be easily elicited with mild external pressure such as a tourniquet test (81). Some patients are affected with a long-term renal inflammation or arthritis after recovery of purpura. Also, it has often been observed that the initiation of joint symptoms is related to minor physical trauma at weight-bearing joints such as the knee and hip in many patients with JIA or other adult arthropathies including osteoarthritis (82). These findings suggest that in Henoch-Schönlein purpura and JIA, the affected target cells are vulnerable to cell injury and induce inflammation as a result of mild physical trauma. It is expected that avoidance of trauma on joints is helpful in part for reducing morbidity and possibly prevention of JIA.

## Immune systems in JIA

Current immunological models about infectious diseases including COVID-19, and infection-associated immune-mediated diseases, including JIA and KD, have limitations to explain the pathophysiology of disease because they are based on the idea that host cell injury is caused by pathogens. In infectious diseases, the viruses or bacteria themselves are not direct toxins to the host cells, but the smaller substances, including PAMPs and DAMPS, produced by infectious insults are responsible for inflammation and subsequent cell injury (12, 78, 83, 84).

Multicellular organisms, including human beings, have evolved from a single-cell organism. Therefore, the immune system of various organisms may have evolved to protect their cells at the molecular biological level. Mammals are composed of numerous different organ-cell types and their own species-specific microbiota. The infected cells by invading microorganisms in microbiota or physically injured cells can produce countless immunologically active substances including pathogenic proteins/peptides, suggesting that the adaptive immune systems of mammals might have evolved mainly against internal insults. Given that immune systems in mammals have evolved to protect self-cells from exposure to the toxic cellular components from other self-cells, it is possible that one of the major functions of apoptosis, autophagy, DNA traps of immune cells, and epigenetic changes such as gene methylation and micro-RNAs within cells perform this critical role at least in part (12). The immune system of mammals, especially adaptive immune systems, matures gradually after birth and progresses into decline in old age (85). The level of white blood cells with neutrophil/lymphocyte differential and values of IgG, IgM, IgA, and IgE differ according to ages in childhood. Because clinical phenotype of the disease is dependent on the host's immune status, disease severity in younger children can differ compared to older children or adults. Infants and young children with infectious diseases such as hepatitis A, coronavirus-associated severe acute respiratory syndrome (SARS), COVID-19, or *Mycoplasma pneumoniae* pneumonia, show milder phenotype compared to older children and young adults (84, 86). Also, majority of patients with chronic immune-mediated diseases in early childhood, including transient neutropenia of infancy, transient erythroblastopenia of childhood, transient hypogammaglobulinemia of infancy, and childhood immune thrombocytopenic purpura, childhood asthma (transient wheezer), and infantile atopic dermatitis show a self-limited clinical course over time. While older children and young adults affected with these diseases show more severe symptoms and more tendency of becoming chronic trait (87, 88). A well-designed study regarding the outcome of JIA reported that with contemporary treatments, the probability of attaining inactive disease exceeded 70% within 2 years in all categories, except for RF-positive polyarthritis (48%), and many patients could discontinue treatment. The probability of attaining remission within 5 years was 46%–57% across JIA categories except for polyarthritis (0% RF-positive, 14% RF-negative) (89). The finding suggests that a larger part of patients with JIA have a self-limited or milder clinical phenotype along with maturing immune function over time, and a part of patients have traits of ongoing chronic arthritis with intermittent relapses, which can continue to adulthood such as RF-positive RA. A small number of patients with JIA, especially systemic symmetric polyarthritis, experience rapidly progressive arthritis with joint tissue destruction within 6 weeks before JIA diagnosis. This type of JIA and/or systemic JIA may be related to the cytokine storm which is associated with excessive immune cell activation against large amount of the etiological substances in the early stage of the diseases as well as other organ diseases caused by infections or non-infectious conditions such as ARDS, extensive myocarditis, rapidly progressive glomerulonephritis, fulminant hepatitis, necrotizing pancreatitis, extensive epidermolysis, acute encephalopathies, and systemic allergic reactions such as anaphylaxis (80, 83, 84, 90). Therefore, as soon as possible, aggressive treatment with systemic immune modulators, including enough-dose corticosteroids and/or intravenous immunoglobulin, is crucial to reduce morbidity and prevent permanent joint malfunctions.

The lower positive rates of RF, antinuclear antibodies, and anti-cyclic citrullinated peptide antibodies are observed in polyarthritis or oligoarthritis in JIA as compared with adults (3, 91, 92). Since the production of these antibodies require the exposed antigens from injured self-cells, increased level of these autoantibodies could reflect a degree of tissue cell injury, active adaptive immune functions, possibly severity of clinical symptoms, and ongoing inflammation. On the other hand, the autoantibodies can be detected in healthy persons and patients with complete remission with high level and long-term, suggesting that the autoantibodies are not pathogenic substances in the diseases. The autoantibodies may be acting against protein antigens derived from self-cells, which could be toxic to other host target cells in the PHS hypothesis (90). In general, children with a maturing adaptive immune system may have less severe clinical phenotypes and more favourable prognoses than adults with arthritis of JIA criteria because maturing of adaptive immune system over time could control the insults from the diseases.

## An immunopathogenesis of JIA through the PHS hypothesis

We have proposed the PHS hypothesis for unifying the pathophysiology of the diseases based on the inductive/deductive reasonings. The diseases include infectious diseases such as influenza, COVID-19 and pneumonia/ARDS (12, 80, 83, 84), and infection-related immune-mediated diseases such as KD and MIS-C (11, 13), and organ diseases such as kidney diseases and central nervous system (CNS) diseases including genetic diseases, prion diseases and Alzheimer disease (93, 94), and cancers (93, 95), and allergic diseases (90). In brief, all diseases have etiological substances that cause the disease, and the immune system, which is part of the PHS, regulates these substances according to their size and biochemical properties for protection of its own cells of the host. The PHS also regulates intracellular or systemic protein deficiencies at least in part. The adaptive immune system controls pathogenic protein/peptide substances and the innate immune system control small non-protein/peptide substances and larger substance such as virions, bacteria, and apoptotic and necrotic bodies. The PHS hypothesis proposes that the phenotypes of each disease, including JIA subtypes and other autoimmune diseases, are determined by the kinds and amounts of etiological substance or events, the target cells in organs, which have an affinity for the substances, and the corresponding immune reactions against the substances. As for autoimmune diseases, the injury of target cells is not caused by specific antibodies or specific T-cell clones against self-antigens expressed on the target cells. Instead, owing to delayed production or lack of specific immune components, persistent abnormal or hyperactive reactions of nonspecific immune components to the substances originated from injured self-cells are responsible for disease progression. Limitations of current immunological models for the diseases and detailed aspects of PHS hypothesis are described in recently published articles (78, 90, 94, 95). The immune components against etiological substances are shown in Table 2 [from reference (90)].

**Table 2:** Immune components in diseases, including JIA, under the PHS hypothesis.

Immune components and effectors (or events)	Main functions and etiological substances
Adaptive immune system	
T cells	Control of pathogenic peptides targeted to host cells, including cancer cells, through production of cytokines (possibly peptides) and immune effectors within the cells in the MHC-restricted (TCR-associated) and non-MHC-restricted events
B cells	Control of pathogenic proteins targeted to host cells, including cancer cells, by production of antibodies in the MHC-restricted (BCR-associated) and non-MHC-restricted events
Innate immune system	
Natural killer cells	Control of transformed cells such as virus-infected cells and tumor cells through their recognizing receptors and immune effectors within the cells
Tissue macrophage-linaeged cells	Antigen presentation to adaptive immune cells in the MHC-restricted immune responses. Possible control of communications between immune cells and affected organ cells including cancers cells in TMEs (95)
Phagocytes (neutrophils and circulating monocyte/macrophages)	Control of large complex substances such as viruses, bacteria, parasites, and apoptotic & necrotic bodies associated with cell injury caused by infection, trauma or other conditions.
Mast cells, basophils, eosionphils	These cells are activated by mainly external toxic substances that are recognized by receptors of the cells and control the substances through immune effetors within the cells and inflammation involved in these cells (90).
Unidentified innate immune components against small non-protein toxic materials	There are non-protein toxic or inflammation-inducing substances, including elements, monoamins, neuropeptides, LPS, RNAs, DNAs, chemicals and biochemicals. TLR-associated immune responses, natural antibodies, and immun proteins and/or peptides control these diverse substances. The immune proteins, including PrP gene products and amyloid proteins, control pathogenic monoamine metabolites or neuropeptides especially in CNS (94).
Production of alternative proteins in genetic diseases and cancer	The systemic and intracellular PHS control in part insults from a protein deficiency or malfunctional protein in organ tissues or within a cell (93–95)

MHC, major histocompatibility complex; TCR, T cell receptor; BCR, B cell receptor; TME, tumor microenvironment; LPS: lipopolysaccharide; TLR, Toll-like receptor; PrP, prion; CNS, central nervous system.

The epidemiological characteristics of JIA such as markedly different incidence among ethnic groups and age predilection in some subtypes are like infection-related immune-mediated disease such as KD or MIS-C. Additionally, clinical and laboratory findings in patients with JIA showing a rapidly progressive arthritis in the acute stage or systemic JIA overlap to those of severe KD, macrophage activation syndrome, or MIS-C (13, 96, 97). Thus, pathogens of JIA, especially systemic JIA, could be presumed as certain strains in microbiota or external pathogens that have adapted as normal flora as with KD or MIS-C (13). The pathogens might be intracellular microbials such as viruses, bacteria or other species that can do intracellular invasion, replication, and production of various etiological or inflammation-inducing substances. Among the strains of normal flora of human species, there may be continuous competition between the host's strains and the strains from other human hosts. It is possible that during the competition, the strains from other persons colonize predominantly and more easily invade the host through the dysbiosis state. Invaded pathogens enter the cells and establish a focus elsewhere in the host. The focus of initial infected cells may locate interface sites of the host where the secondary lymphoid organs (e.g., tonsils, regional lymph nodes, or Peyer's patches) are distributed for control of invading pathogens. The focus as a localized lesion provides pathogen-origin and host-cell-origin materials which have various sizes and biochemical properties. Each corresponding immune component of innate immune system cells, including neutrophils and macrophages, and immune proteins and possibly immune peptides, and adaptive immune components work for control of the substances. Most of infected patients recover from the initial pathogen infections as localized inflammation with few symptoms of arthritis. While some infected patients develop arthritis when arthro-toxic substances spread into systemic circulation and bind to target cells in joints, including synoviocytes, chondrocytes, and/or connective tissue cells, and clinical symptoms begin to appear. If pathogenic peptides and/or proteins attach to the receptor of target cells in joints, nonspecific T cells and B cells (antibodies) make up the first-line effectors against the substances, since specific T-cell clones and antibodies (B cells) against pathogenic peptides/proteins require several days or more owing to the immune gene recombination of TCRs and BCRs (90). During these pathologic or non-specific processes, cytokine imbalance such as cytokine storm is involved in the target cell injury although the mechanisms of cytokine storm remain unknown. Also, affected target cells could become vulnerable to mild traumatic injury at this stage of JIA, and substances derived from physically injured target cells induce more inflammation. After the appearance of specific immune components, the inflammatory substances, including the pathogenic peptides/proteins, are effectively controlled and inflammation processes cease. The immune system of patients with JIA may delay or be unable to induce specific clones against pathogenic proteins/peptides derived from injured cells, while the immune system of most of patients with postinfectious arthritis has this ability and induces early recovery from arthritis. The ongoing activation of nonspecific adaptive immune cells with high levels of proinflammatory cytokines may be responsible for further injury of target cells and neighbouring cells, and cells of remote organs on occasion. Thus, genetically determined immune status (the repertoires of T-cell and B-cell clones and other immune components) in each person decide the prognosis of JIA, other autoimmune diseases, and chronic inflammatory diseases (Figure 1).

## Conclusions

JIA has heterogeneous subtypes with different incidence, age, and sex in each subtype, and the etiology and pathophysiology of JIA remain to be proven. We herein discussed the epidemiological and clinical characteristics of JIA, including the different incidence among the ethnic groups, the association with infections, other disease conditions and physical trauma, and the different clinical nature as compared with arthritis in adults. We proposed a presumed etiology and immunopathogenesis of JIA based on these characteristics of the disease and the PHS hypothesis. We hope that this article helps researchers and clinicians to resolve the puzzles remaining regarding the etiology, pathophysiology, and treatment of JIA.
